# Extracts from the Proceedings of the Wayne County Medical Society

**Published:** 1861-05

**Authors:** 


Extracts from the Proceedings of the Wayne County Medical Society.—*Position a Remedy for Stertor.*—Mr. Bowles, after repeated trial, assures the profession that stertor in apoplexy, etc., is immediately relieved by turning the patient well on his side, so that the paralyzed tongue and velum palati will fall forward, and the mucus drain away. Very soon, also, the phenomenon of partial suffocation accompanying the stertor, will disappear.

*Acupressure as an Hæmostatic.*—Originating with Professor Simpson, this method of arresting surgical hemorrhage in some cases has been warmly advocated by some good surgeons, and condemned by others. Its real value will only be established after further experience.

*Fat as an Antidote to Poisoning by Arsenic.*—A small quantity of fat, as of milk, meat, etc., will reduce the solubility of arsenious acid to about one-twentieth of what ordinarily belongs to it, according to Dr. Blondlot. Hence, fat swallowed immediately after arsenic, will hold it nearly or quite harmless until radical means can be adopted to evacuate the stomach. If this is true, it has immense practical value.—*Lancet and Observer.*

